# Identification and Characterization of microRNAs during Maize Grain Filling

**DOI:** 10.1371/journal.pone.0125800

**Published:** 2015-05-07

**Authors:** Xining Jin, Zhiyuan Fu, Panqing Lv, Qian Peng, Dong Ding, Weihua Li, Jihua Tang

**Affiliations:** National Key Laboratory of Wheat and Maize Crop Science, Collaborative Innovation Center of Henan Grain Crops, College of Agronomy, Henan Agricultural University, Zhengzhou, 450002, China; Kunming University of Science and Technology, CHINA

## Abstract

The grain filling rate is closely associated with final grain yield of maize during the period of maize grain filling. To identify the key microRNAs (miRNAs) and miRNA-dependent gene regulation networks of grain filling in maize, a deep-sequencing technique was used to research the dynamic expression patternsof miRNAs at four distinct developmental grain filling stages in Zhengdan 958, which is an elite hybrid and cultivated widely in China. The sequencing result showed that the expression amount of almost all miRNAs was changing with the development of the grain filling and formed in seven groups. After normalization, 77 conserved miRNAs and 74 novel miRNAs were co-detected in these four samples. Eighty-one out of 162 targets of the conserved miRNAs belonged to transcriptional regulation (81, 50%), followed by oxidoreductase activity (18, 11%), signal transduction (16, 10%) and development (15, 9%). The result showed that miRNA 156, 393, 396 and 397, with their respective targets, might play key roles in the grain filling rate by regulating maize growth, development and environment stress response. The result also offered novel insights into the dynamic change of miRNAs during the developing process of maize kernels and assistedin the understanding of how miRNAs are functioning about the grain filling rate.

## Introduction

As one of the most important grain crops and also a source of feed, food and fuel, maize (*Zea mays* L.) is cultivated broadly in the world. The maize yield is mainly determined by kernel weight and kernel numbers, among which the kernel weight is affected by the grain filling rate and duration [1]. During the period of maize grain filling, the grain filling rate is closely associated with kernel weight [2]. The genetic variability in plant senescence and grain filling rates needs to be exploited to help stabilize the component of yield [3]. It has been shown that the growth rate increased with rising temperatures [4] and that the decrease of gibberellins or the increase of abscisic acid could enhance the remobilization of carbon to the grains and promote the grain filling rate [5]. Some quantitative trait loci [6] and important proteins [7] have also been identified, which have contributed much to the grain filling rate in maize.

miRNAs are fundamental, sequence-specific regulatory elements of eukaryotic genomes. In plants, these 19–24 nucleotide (nt)-long RNA species mediate the expression of endogenous genes at the transcriptional and post-transcriptional levels [8]. The near-perfect or perfect complementarity between the sequence of plant miRNAs and their targets suggests that most of the plant miRNAs have a similarly function with small interfering RNAs [9]. Research analyzing the spatial expression of miRNAs has shown that miRNAs have a tissue-specific expression during plant development [10], which indicates that miRNAs are possibly involved in specifying and maintaining tissue identity. miRNAs play an crucial role in many biological processes of maize, including leaf development [11, 12], root development [13], seed germination [14] and response to abiotic stresses [15, 16]. The results of deep sequencing showed that miRNAs also affect the rice grain filling [17–19].

Measuring the proposed grain filling rate is not easily integrated into many studies because the grain filling rate is an environmentally modified quantitative phenotype [20–22]. Generally, a complicated character is difficult to advance directly, but perhaps it will be more easily to adopt the indirect selection way [2]. From the perspective of reverse genetics, proteomic study has been used to identify special proteins which associated with the grain filling stage and explore the main factors which affected the grain filling rate of maize [7]. Identifying the expression quantity of miRNAs in different grain filling stages would also be important in identifying the miRNA-dependent gene expression regulatory networks of maize grain filling.

Compared with inbred lines, hybrid maize genotypes have a larger cultivated area and more grain yield worldwide. Therefore, using hybrids to inspect the molecular mechanisms of the grain filling rate, instead of inbred lines, is more meaningful for applying genetic manipulations in maize [7]. In this study, the Solexa deep-sequencing technique was performed on four main grain filling stages of an elite maize hybrid, Zhengdan 958, in China. The objectives of this investigation were: 1) to identify maize conserved miRNAs and predict novel miRNAs involved in maize grain filling; and 2) to construct the key miRNA-dependent gene expression regulatory networks of maize grain filling.

## Materials and Methods

### Plant materials

An elite commercial hybrid, Zhengdan 958 (Zheng 58 × Chang 7–2), which has been the most widely planted maize hybrid since 2005 in China, was used as the plant material. The hybrid Zhengdan 958 was planted on 5 May 2011 at the farmland of the Henan Agricultural University (Zhengzhou, China; E113°42', N34°48'), where the average temperature is 14.3°C and the average rainfall is 640.9 mm per year. Two replication plots of the hybrid were planted in the field, of each row the length is 4 m, the inter-row space is 75 cm and the within-row space is 25 cm.

**Fig 1:**
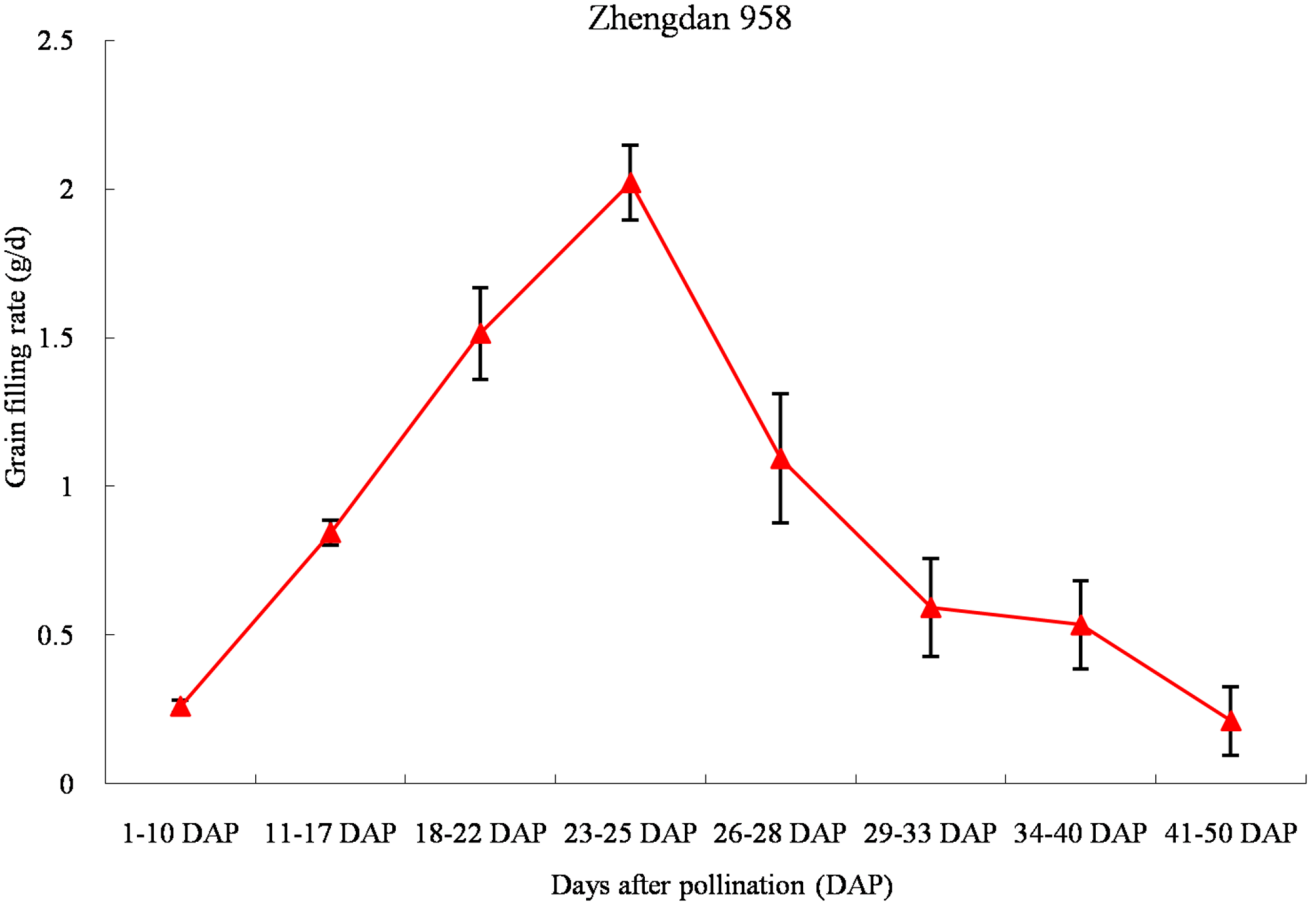
Grain filling rate of the maize hybrid Zhengdan 958 from 10 to 50 days after pollination (DAP).

To achieve a consistent grain filling rate, the plants were all pollinated themselves on 6 July, then the middle kernels of ears from each plot were collected at 10, 17, 22, 25, 28, 33, 40 and 50 days after pollination (DAP). Three hundred kernels from each replication plot were dried at 70°C for 24 hours and the dry weights were measured. Divide the increment of dry weight by the number of days and kernels between two grain filling stages is the grain filling rate (Fig 1). The corresponding kernels were cut to discard the pericarps and embryos, then the endosperms were stored at −80°C for miRNA and mRNA extraction. According to the change of the grain filling rate in the whole grain filling stages, endosperms obtained at 17, 22, 25 and 28 DAP were applied for further miRNA and mRNA analyses.

### RNA extraction, deep sequencing, and data analysis

Total RNA was extracted with the Plant Total RNA Extraction kit (Bioteke Corporation, Beijing, China) by the manufacturer’s method, and 2 g endosperms were used for RNA extraction and deep sequencing.

After removing the low-quality reads from the raw data, the appropriate small RNAs were mapped to the reported miRNAs from miRBase (http://www.mirbase.org/). The expression amount of sequence was measured by RPM (reads per million) to get the comparable expression amounts. After normalization, the miRNA expression amount who was < 1 RPM in all four samples was discarded [18].

The software Mireapwas used to manage the unannotated small RNA reads to identify novel miRNAs. One small RNA was deemed to be a novel miRNA only if it accorded with the strict criteria described by Dong Ding [14]. Only those candidates with a minimal folding free energy index (MFEI) > 0.85 were treated as novel maize miRNAs [14]. The 74 novel miRNAs sequences have been deposited into Genbank, NCBI (http://www.ncbi.nlm.nih.gov/genbank/) with the accession numbers of KP192041-KP192114 (S1 Table).

### Prediction and functional analyses of maize miRNA targets

The potential targets of miRNAs were calculatedby the psRNA-Target software with default parameters [23]. The maize PlantGDB genomic CD library was served as the database for the target searching. The function annotation of Potential miRNA targets were blasted against GO database using AgriGO with default parameters.

### Expression profile of selected miRNAs

Six conserved miRNAs and six novel miRNAs were choosed to establish the sequence results. The reverse transcription and the absence of genomic DNA were performed using the One Step PrimeScript miRNA cDNA Synthesis Kit (TaKaRa, Dalian, China). For doing quantitative real-time PCR (qRT-PCR), the SYBR Premix Ex Taq (Takara, Dalian, China) was used and worked on a Fluorescence detection system (Bio-Rad, Waltham, MA, USA). Primer specificity was verified by the Melt curve analysis. The relative expression quantity of the miRNAs was calculated by the comparative threshold cycle (CT) method. The *tubulin* gene was performed as an inner control to normalize data among samples. Every sample was performed in technical triplicate. The reverse primer of miRNAs used for qRT-PCR is the Uni-miR qPCR primer in miRNA cDNA Synthesis Kit. The other qRT-PCR primers are listed in Table 1.

**Table 1:** qRT-PCR primer sequences formaize (*Zea mays* L.) miRNAs, target genes and *tubulin*.

Primer	Primer sequence (5′-3′)	Primer	Primer sequence (5′-3′)
***Tubulin* (F)**	GCAGAACAAGAACTCGTCCTACT	***Tubulin* (R)**	TCAGCCTCAGTGAACTCCATCT
***GRF(F)***	CAGGGAACTGGTGAACAAACA	***GRF(R)***	ATCAGTGAATCCGATGAAACG
***SPL(F)***	TCGCACCTTCGCTTTACG	***SPL(R)***	ACTGGCCGCCTCATCACTC
***LAC 17(F)***	CAAACCGATACTCCCTGCTCTA	***LAC 17(R)***	GACCTTGGCATGTCTGGTTATT
**zma-miR156**	TGACAGAAGAGAGTGAGCAC	**zma-miRt4**	TGAAGAGAATTGAGGGGGCTA
**zma-miR162**	TCGATAAACCTCTGCATCCA	**zma-miRt13**	TCTAGAATCAAAGGCTAATCT
**zma-miR172**	AGAATCTTGATGATGCTGCA	**zma-miRt15**	ATGCAGAACAATTTACAGACG
**zma-miR393**	TCCAAAGGGATCGCATTGATCT	**zma-miRt17**	TGGTCTTGCCACCGTGTCGTCC
**zma-miR396**	TTCCACAGCTTTCTTGAACTG	**zma-miRt21**	GGATGTCGATATTGGAGGGCA
**zma-miR397**	TCATTGAGCGCAGCGTTGATG	**zma-miRt28**	TTTGGGGTGGATACGTGGTCA
**zma-miR408**	CTGCACTGCCTCTTCCCTGGC		

## Results

### Grain filling rate analysis

The grain filling rate of hybrid Zhengdan 958 was measured at several stages of the grain filling (10 to 50 DAP). The grain filling rate showed a step increase from 10DAP and reached its highest level at 23–25 DAP; after 25 DAP, it decreased rapidly until 33 DAP, and then decreased moderately from 33 DAP to 50 DAP (Fig 1). These results implied that the rapid grain filling rate around 25 DAP might make a huge contribution to the dry matter content and the yield of the hybrid Zhengdan 958.

### The detected miRNAs in four phases of grain filling

The maximum of grain filling rate occurred at 23–25 DAP in Zhengdan 958 (Fig 1), so we chose the four key sampling stages (17 DAP, 22 DAP, 25 DAP and 28 DAP) assayed by Solexa. In total, there were 25 families containing 173 reported maize miRNAs. After removing the miRNAs with low detectability levels (< 1 RPM at all four sampling phases), there were 77 conserved miRNAs belonging to 17 miRNA families co-detected in endosperm at the four sampling times, 17, 22, 25 and 28 DAP. The detected miRNAs in these samples have a significant variety in relative expression abundance, with over 70,000 RPM of miR168 and 1 RPM of miR162 in the 28 DAP sample. These different expression amounts of miRNAs in the grain filling duration meant that the miRNA target genes may be post-transcriptionally changed in the grain filling of maize. Furthermore, it is not that all the conserved miRNA family members were detected, and the expression levels among miRNA families were different (Table 2), which suggested that the miRNAs might have tissue- or developmental stage-specific expression patterns.

**Table 2:** Expression levels and trends for miRNAs detected in maize grain filling.

miRNA	17DAP RPM	22DAPRPM	25DAPRPM	28DAPRPM	Changing pattern
zma-miR156^a^	5476.13	8222.89	10448.19	11040.79	a
zma-miR156^b^	5476.13	8222.89	10448.19	11040.79	a
zma-miR156^c^	5476.13	8222.89	10448.19	11040.79	a
zma-miR156^d^	5477.80	8226.20	10457.58	11046.46	a
zma-miR156^e^	5476.13	8222.89	10448.19	11040.79	a
zma-miR156^f^	5476.13	8222.89	10448.19	11040.79	a
zma-miR156^g^	5476.13	8222.89	10448.19	11040.79	a
zma-miR156h	5476.13	8222.89	10448.19	11040.79	a
zma-miR156i	5476.13	8222.89	10448.19	11040.79	a
zma-miR156j	576.34	950.15	1452.05	822.86	c
zma-miR156k	12.70	21.55	29.90	71.45	a
zma-miR156l	4651.30	6617.51	7033.87	8192.08	a
zma-miR159a	180.67	95.98	241.08	77.12	g
zma-miR159^b^	180.67	95.79	239.91	76.91	g
zma-miR159^f^	180.67	95.98	240.83	77.12	g
zma-miR159j	180.67	95.79	239.66	76.91	g
zma-miR159k	180.67	95.79	239.66	76.91	g
zma-miR162	0.28	0.28	0.74	1.44	a
zma-miR164a	10.06	4.17	9.51	6.80	g
zma-miR164^b^	10.06	4.17	9.51	6.80	g
zma-miR164^c^	10.06	4.17	9.51	6.80	g
zma-miR164^d^	10.06	4.17	9.51	6.80	g
zma-miR164^e^	1231.66	1126.67	1062.36	2886.00	d
zma-miR164^g^	10.06	4.17	9.51	6.50	g
zma-miR166a	4232.75	4676.96	8108.95	8397.87	a
zma-miR166^b^	4232.48	4676.49	8108.15	8396.95	a
zma-miR166^c^	4232.48	4676.86	8108.39	8397.15	a
zma-miR166^d^	4232.48	4676.49	8108.15	8396.95	a
zma-miR166^e^	4232.48	4676.49	8108.15	8396.95	a
zma-miR166^f^	4192.62	4649.96	8120.75	8540.57	a
zma-miR166^g^	4191.05	4647.68	8112.47	8528.09	a
zma-miR166h	4192.62	4649.96	8120.75	8540.57	a
zma-miR166i	4232.11	4675.44	8106.67	8395.09	a
zma-miR166j	29.07	0.47	1.05	1.03	g
zma-miR166k	29.07	0.47	1.05	1.03	g
zma-miR166l	259.93	222.47	361.31	333.54	g
zma-miR166m	154.28	118.34	217.31	158.98	g
zma-miR166n	29.07	0.47	1.05	1.03	g
zma-miR167a	319.08	169.51	307.71	284.97	g
zma-miR167^b^	311.42	169.89	308.70	285.90	g
zma-miR167^c^	319.08	169.51	307.71	284.97	g
zma-miR167^d^	319.08	169.51	307.71	284.97	g
zma-miR167^e^	6587.38	8103.41	11751.23	12720.22	a
zma-miR167^f^	6600.21	8122.36	11772.78	12742.91	a
zma-miR167^g^	15643.25	1732.79	1899.07	3669.20	f
zma-miR167h	15621.20	1730.99	1900.31	3662.91	f
zma-miR167i	15621.20	1730.99	1900.31	3662.91	f
zma-miR167j	6633.98	8177.50	11858.06	12818.38	a
zma-miR168a	35427.44	49707.89	61008.07	70041.70	a
zma-miR168^b^	35427.44	49707.89	61008.07	70041.70	a
zma-miR169o	15.32	6.06	16.67	28.46	f
zma-miR171^b^	228.38	246.35	188.59	128.16	e
zma-miR171^d^	228.19	247.01	188.90	128.26	e
zma-miR171^e^	228.19	247.01	188.90	128.26	e
zma-miR171^f^	228.38	246.35	188.59	128.16	e
zma-miR171i	228.38	246.63	188.90	128.16	e
zma-miR171j	229.39	247.20	189.95	129.19	e
zma-miR172a	13.56	3.03	5.25	2.27	g
zma-miR172^b^	13.56	3.03	5.25	2.27	g
zma-miR172^c^	13.56	3.03	5.25	2.27	g
zma-miR172^d^	13.56	3.03	5.25	2.27	g
zma-miR393a	7.94	2.56	5.99	7.42	f
zma-miR393^b^	1.02	0.28	0.37	0.93	f
zma-miR393^c^	7.94	2.56	5.87	7.22	f
zma-miR396a	6.83	7.49	8.27	11.55	a
zma-miR396^b^	6.83	7.49	8.27	11.55	a
zma-miR396^c^	19.56	14.78	15.69	25.26	f
zma-miR396^d^	19.56	14.78	15.69	25.26	f
zma-miR397a	1.08	0.86	0.66	0.18	b
zma-miR397^b^	11.90	8.73	6.66	2.45	b
zma-miR398a	3.23	2.46	3.21	2.47	g
zma-miR398^b^	3.23	2.46	3.21	2.47	g
zma-miR408	25.84	10.80	27.11	12.17	g
zma-miR408^b^	25.84	10.80	27.11	12.17	g
zma-miR528a	8120.22	7756.05	4760.55	3778.28	b
zma-miR528^b^	8191.09	7803.33	4796.74	3804.06	b
zma-miR827	354.97	53.63	139.81	112.69	g

^a^, miRNAs whose abundance increased linearly from 17 to 28 DAP;

^b^, miRNAs whose abundance decreased linearly from 17 to 28 DAP;

^c^, miRNAs up-regulated at 25 DAP;

^d^, miRNAs down-regulated at 25 DAP;

^e^, miRNAs up-regulated at 22 DAP;

^f^, miRNAs down-regulated at 22 DAP;

^g^, miRNAs that had irregular changes.

The MFEI is an important criterion for identifying miRNAs from other smallRNAs. In this study, the newly identified maize pre-miRNAs had high MFEI values (0.87–2.36; S1 Table), with an average of about 1.31 [24]. Seventy-four novelmiRNA candidates belonging to 33 families were co-detected at the four sampling times (S1 Table). The novel miRNAs were all expressed at low levels, with 84 RPM being the highest relative abundance. This is consistent with the study in rice grain filling [17].

### The expression patterns of miRNAs during maize grain filling

Through the deep sequencing, the expression amount of almost all miRNAs was found changing with the development of the grain filling. The detected RPM of the 77 co-detected conserved miRNAs and 74 novel miRNAs were revealed in seven groups (Table 2 and S1 Table). Except for the irregular group (group g), Group I increased linearly from 17 to 28 DAP formed the largest group, represented by 28 out of 77 of the conserved miRNAs. The following group was down-regulated at 22 DAP (group f) and represented 9 out of 77 miRNAs. In novel miRNAs, those up-regulated at 25 DAP (group c) formed the largest group (17/74). The dynamic expression of these miRNAs, such as group c and group d were closely related with the variation of grain filling rate, might play important roles in controlling many biological processes during maize grain filling by cleaving the transcript of their corresponding target genes or repressing the translation of these genes.

### Functional analysis of the target genes of the detected miRNAs

**Fig 2:**
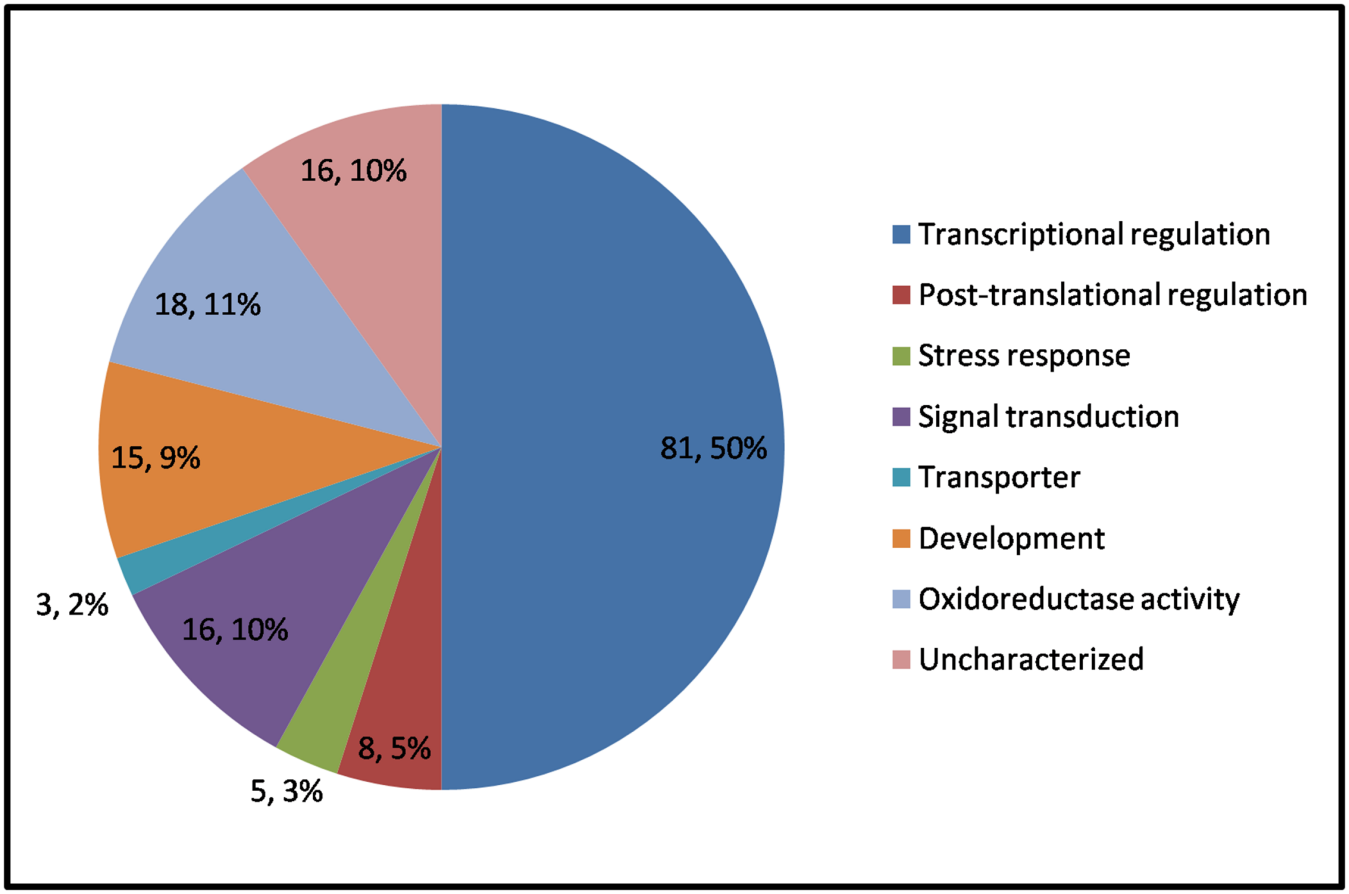
Functional classification of conserved maize miRNA targets.

The target genes of the 77 conserved miRNAs and 74 novel miRNAs were predicted with the psRNA Target tool. A total of 162 and 160 miRNA targets were detected from the conserved and novel miRNAs, respectively. The function of these target genes were further annotated by GO program. Only a small part of the novel miRNA targets produced GO results, including signal transduction-related genes and some organelle-specific genes (S2 Table). On the basis of functional annotations, the 162 target genes of the conserved miRNAs (S3 Table) were classified into eight groups (Fig 2), transcriptional regulation (81, 50%), oxidoreductase activity (18, 11%), signal transduction (16, 10%), development (15, 9%), post-translational regulation (8, 5%), stress response (5, 3%), transporter (3, 2%) and uncharacterized (16, 10%).

### Expression profiles of selected miRNAs and target genes

**Fig 3:**
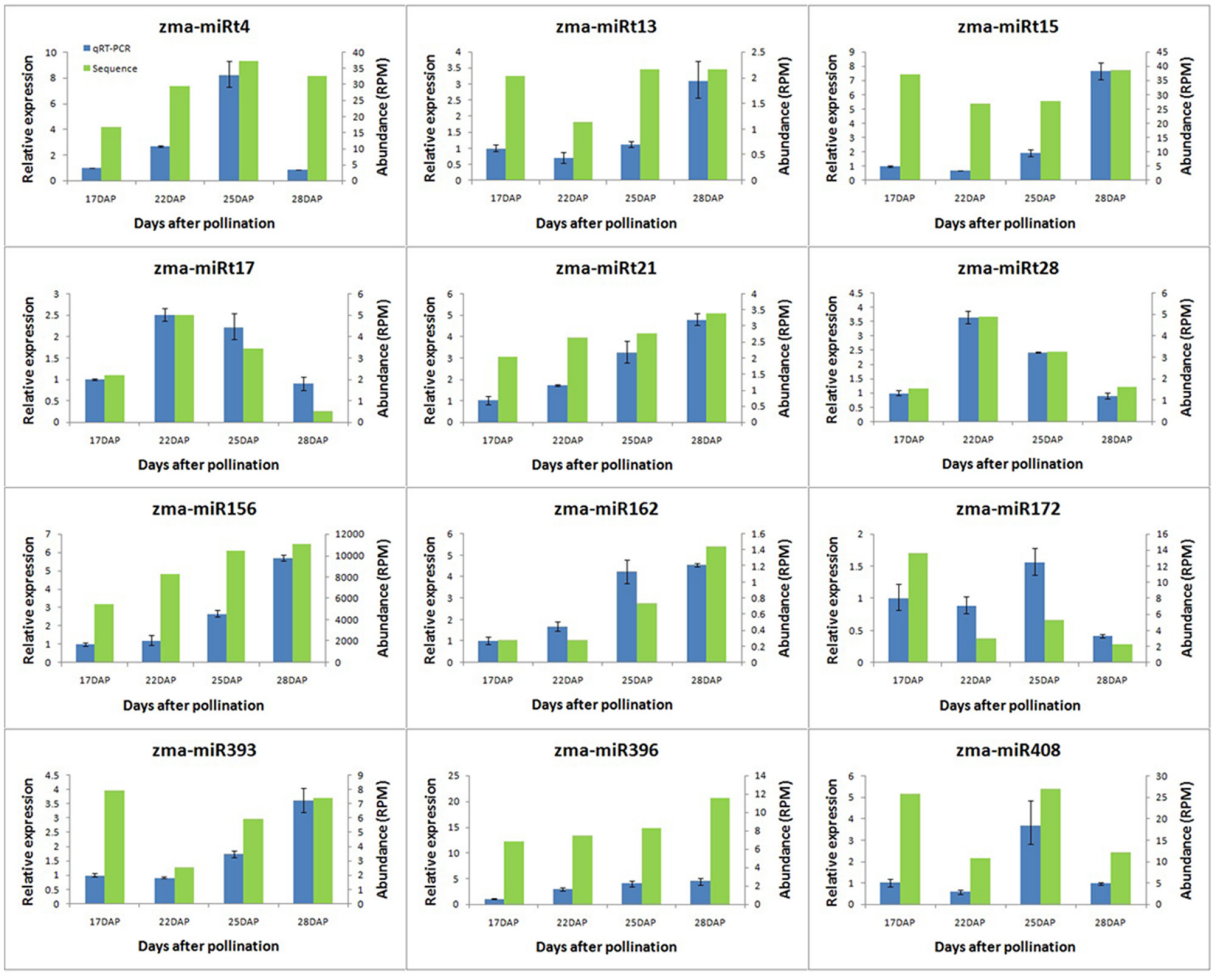
Relative expression amounts and the abundance of selected miRNAs sequenced during maize grain filling. RPM represents reads per million and DAP represents days after pollination.

**Fig 4:**
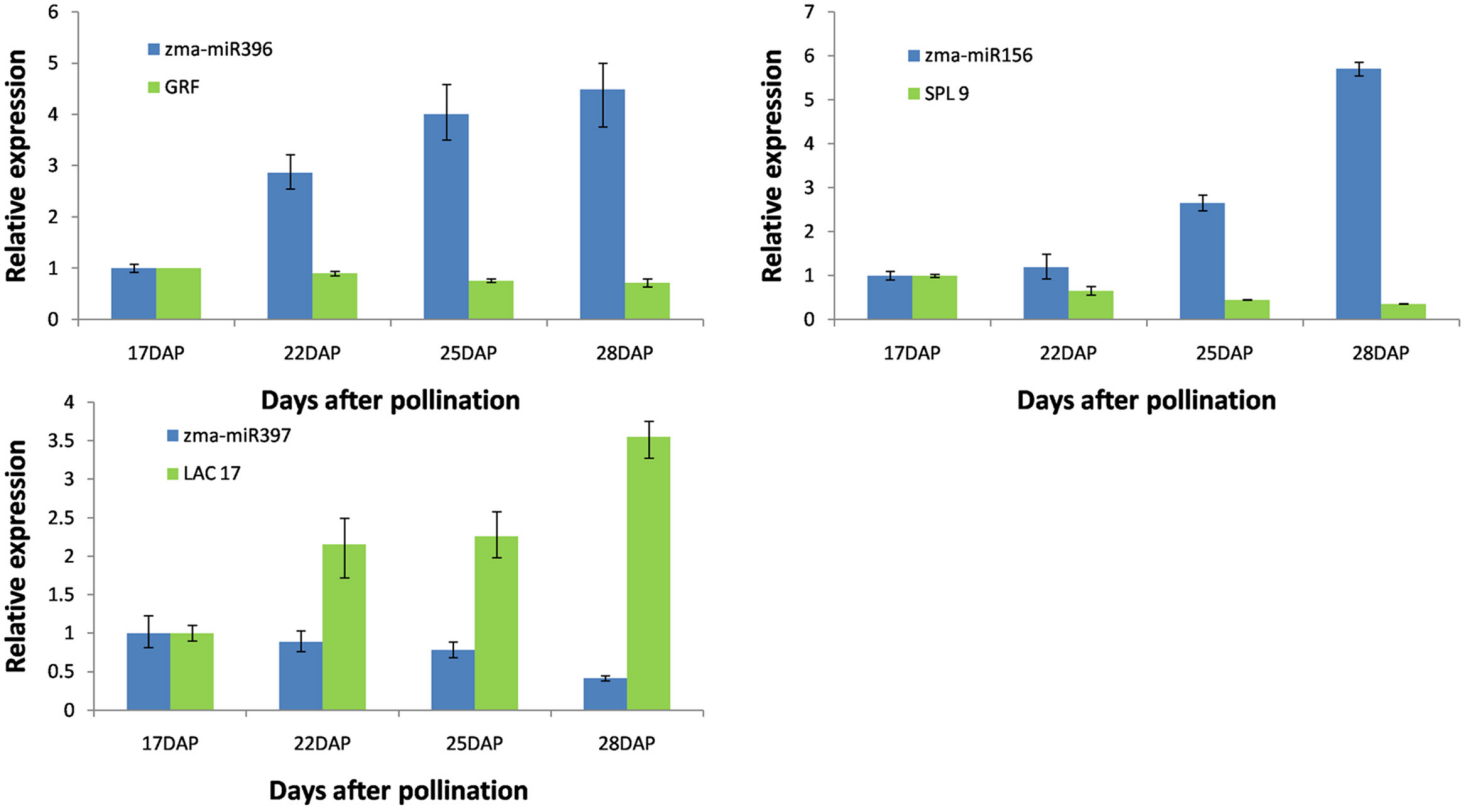
Relative expression levels of three key maize miRNAs and their target genes. DAP represents days after pollination.

Six novel maize miRNAs (miRt4, miRt13, miRt15, miRt17 and miRt21, miRt28) and six conserved miRNAs (miR156, miR162, miR172, miR393, miR396 and miR408) were chosen to verify the sequencing results via the qRT-PCR analysis. These results revealed that the relative expression levels of selected miRNAs in grain filling corresponded to the deep-sequencing data (Fig 3). Of the target gene expression profiles detected, three key miRNA target genes showed opposite expression trends compared with the miRNAs (Fig 4), but the other target genes did not show a clearly opposite expression profile (data not shown). These results may suggest that the expression of these target genes was influenced by other factors in addition to the corresponding miRNAs.

## Discussion

### Key period of the grain filling

The grain filling rate directly determines the final grain weight, and it is a key component of the total grain yield. By comparing 10 cultivars of spring wheat’s grain yield measured at intervals after anthesis, Nass and Reiser [25] found that the rate of the grain filling was a key factor in determining the final grain weight; however, the duration of the grain filling period was not an important factor. Ignoring the genetic background, in maize inbred lines and hybrids, the maximum of grain filling rate occurs between 21 and 25 DAP [26]. It was also found that the difference of maize kernel weight was mostly decided by the grain filling rate from 16 DAP to 29 DAP [6]. In our research, the maximum of grain filling rate occurred at 25 DAP in Zhengdan 958 (Fig 1). So the study on the four key sampling stages (17 DAP, 22 DAP, 25 DAP and 28 DAP) around 25 DAP by Solexa, which maybe detect many miRNAs that had important effects on the development of maize kernels.

### miRNAs function on transcriptional regulation during maize grain filling

miRNAs have especially important roles in controlling plant development by regulating many transcription factor genes at the post-transcriptional level [27]. For example, the *Squamosa promoter binding protein-like* (*SPL*) familyis considered to be the target gene of zma-miR156. Ten out of the 16 *SPL* family members have been predicted to be miR156 targets [9]. *SPL3*, *SPL4*, *SPL5* and *SPL9* make a secondary contribution to the regulation of flowering [28, 29] and appear to function mostly in the control of flowering time and phase change [28]. A point mutation within the miR156 target regin of *OsSPL14* in rice generates the plant with a reduced tiller number, increased lodging resistance and enhanced grain yield [30]. The other result also showed that the higher expression of *OsSPL14* could lead to increased primary branch number in panicles, and then an increase in rice yield [31]. As another target gene of miR156, *OsSPL16* encodes a protein that promotes cell division, also with positive consequences for grain width and yield in rice [32]. In the present study, zma-miR156 increased linearly from 17 to 28 DAP, this is consistent with the result in the seed development process of rice [33], wheat [34] and barley [35]. We also detected the expression pattern of *SPL*, and found that *SPL* reduced linearly with the increase of zma-miR156 (Fig 4). Taken together, the higher expression of *SPL* in the early stage maybe can increase the width and the number of the kernal, and with the development of kernals the lower level of *SPL9* and other related genes might prolong the phase associated with the rapid grain filling rate.

miR396 regulates *growth-regulating factors* (*GRFs*), which are transcription factors of the plant-specific family. In the leaf primordia, miR396 expresses at low levels throughout the meristem, overlapping with the expression of its target, *GRF2* [36]. Over-expression of miR396 was found to decrease the *GRFs* that has been shown to influence cell proliferation in the meristem and developing leaves [37]. Most of the *GRF* genes are expressed in actively growing tissues, such as shoot tips and flower buds, but weakly in mature stems and leaf tissues [38]. In this study, the results showed that zma-miR396 increased in the endosperm at the four sampling times, but the expression level was weak, with values less than 30 RPM. During the seed development process of wheat and rice, the expression amount of miR396 was weak too, some of them even less than 1 RPM [33, 34].The qRT-PCR results revealed that *GRF* decreased a little with a slight increase of zma-miR396 (Fig 4). The repression of zma-miR396 maybe leads to the steady expression amount of *GRFs*, and enhances the development of the corn kernel.

### miRNA involved in regulating oxidoreductase activity during maize grain filling

Oxidoreductases are enzymes that catalyze the transfer of electrons from one molecule to another, with a slow release of energy. Many oxidoreductases play key roles in plant development, such as *NADH-ubiquinone oxidoreductase* [39], *oxalate oxidoreductase* [40] and *xanthine oxidoreductase* [41]. Lu et al. (2013) characterized the *laccase* gene family and identified 49 *laccase* genes, of which 29 were predicted to be targets of *Populustrichocarpa* (ptr)-miR397a [42]. Laccases are often able to catalyze oxidation of lots of substrates, such as phenols and amines [43]. It was also reported that plant laccases assist in wound healing [44], and in forming seed coat cell walls [43]. There are some other evidence showed that overexpression of miR397 can improve rice yield, mainly by increasing the grain size (result from cell division but not cell expansion) and promoting panicle branch [45]. But during the seeds development process of many crops, such as wheat [34], rice [33], and maize in the present study, the expression amount of miR397 are all maintaining a very low level. *LAC 17* is the main oxidoreductase gene found in this study, and the qRT-PCR results showed that the relative abundance of *LAC 17* increased slowly with the light decrease of miR397 across the four consecutive grain filling stages (Fig 4). In conclusion, miR397 may play important role in the early time of reproductive stage, but in the later seeds development process it’s the *LAC* genes who supply energy and improve the maize’s ability to resist environmental stresses during the grain filling stage.

### Other miRNAs involved in regulating maize grain filling

**Fig 5:**
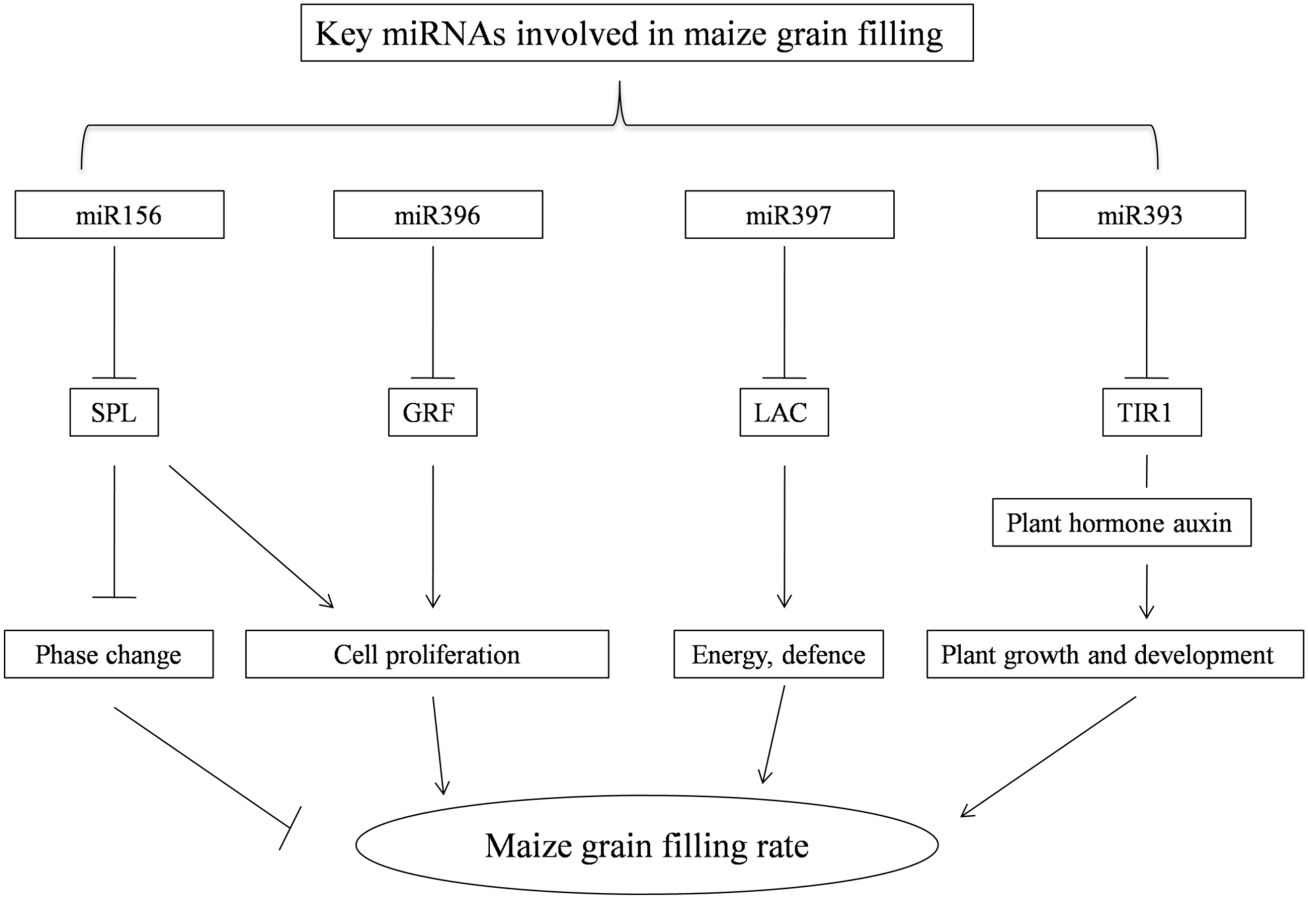
A possible functional network of maizekey miRNAs involved in maize grainfilling. The arrows indicate positive regulation, the nail shapes represent negative regulation, and straight lines indicate regulation. *SPL*: *Squamosa promoter binding protein-like*; *GRF*: *Growth-regulating factors*; *LAC*: *laccase*; and *TRI1*: *Transport inhibitor response 1*.

*Fatty acid desaturases* play an important role in the maintenance of the proper structure and function of biological membranes [46]. In this study, *fatty acid desaturases* were predicted to be targets of zma-miR169o by psRNA-Target software (S3 Table). zma-miR169o was down-regulated at 22 DAP and then increased with the following grain filling stages. Similarly, zma-miR393, which targets the F-box auxin receptor *transport inhibitor response 1* (*TIR1*) [47], was also down-regulated at 22 DAP. The plant hormone auxin regulates diverse aspects of plant growth and development [48]. Studies have shown that *TIR1* is an auxin receptor, which mediated auxin/indole-3-acetic acid protein degradation and auxin-regulated transcription [49]. These miRNAs, together with the miRNAs that function on transcriptional regulation and oxidoreductase activity, are all involved in coordinating the development of maize grain filling (Fig 5).

## Conclusions

This work showed during developmental stages of the maize grain filling, the dynamic characteristics of miRNAs may be meaningful in more precisely learning the regulatory roles of miRNAs. miRNA156, 393, 396 and 397, and their respective targets, may contribute to the maize grain filling rate by regulating maize growth, development and environment stress response (Fig 5). Novel miRNAs were expressed at low levels and the function of the predicted targets was limited (S2 Table).

## Supporting Information

S1 Fig The structure of pre-miRNA156, 169, 393, 396 and 397. (DOCX)

S1 Table The list of novelmaizemiRNAs. (XLSX)

S2 Table The list of target genes for the novelmaizemiRNAs. (XLSX)

S3 Table The list of target genes for the conservedmaize miRNAs. (XLSX)
