# Supplementary figures and images for: Identification and Characterization of microRNAs during Maize Grain Filling
Source: PLoS One. 2015 May 7;10(5):e0125800. doi: 10.1371/journal.pone.0125800 (PMC4423906; doi:10.1371/journal.pone.0125800)

zma-miR156a


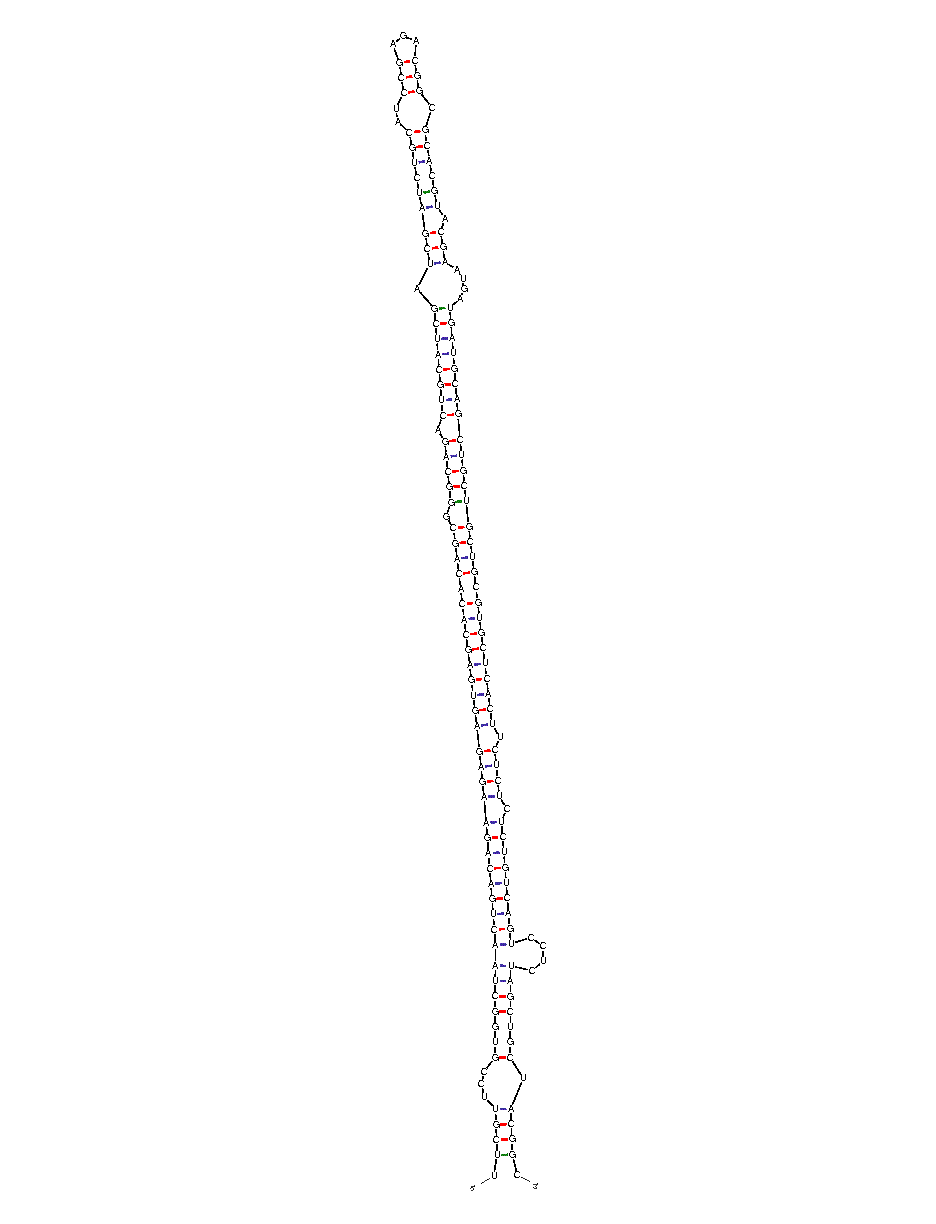


zma-miR169a


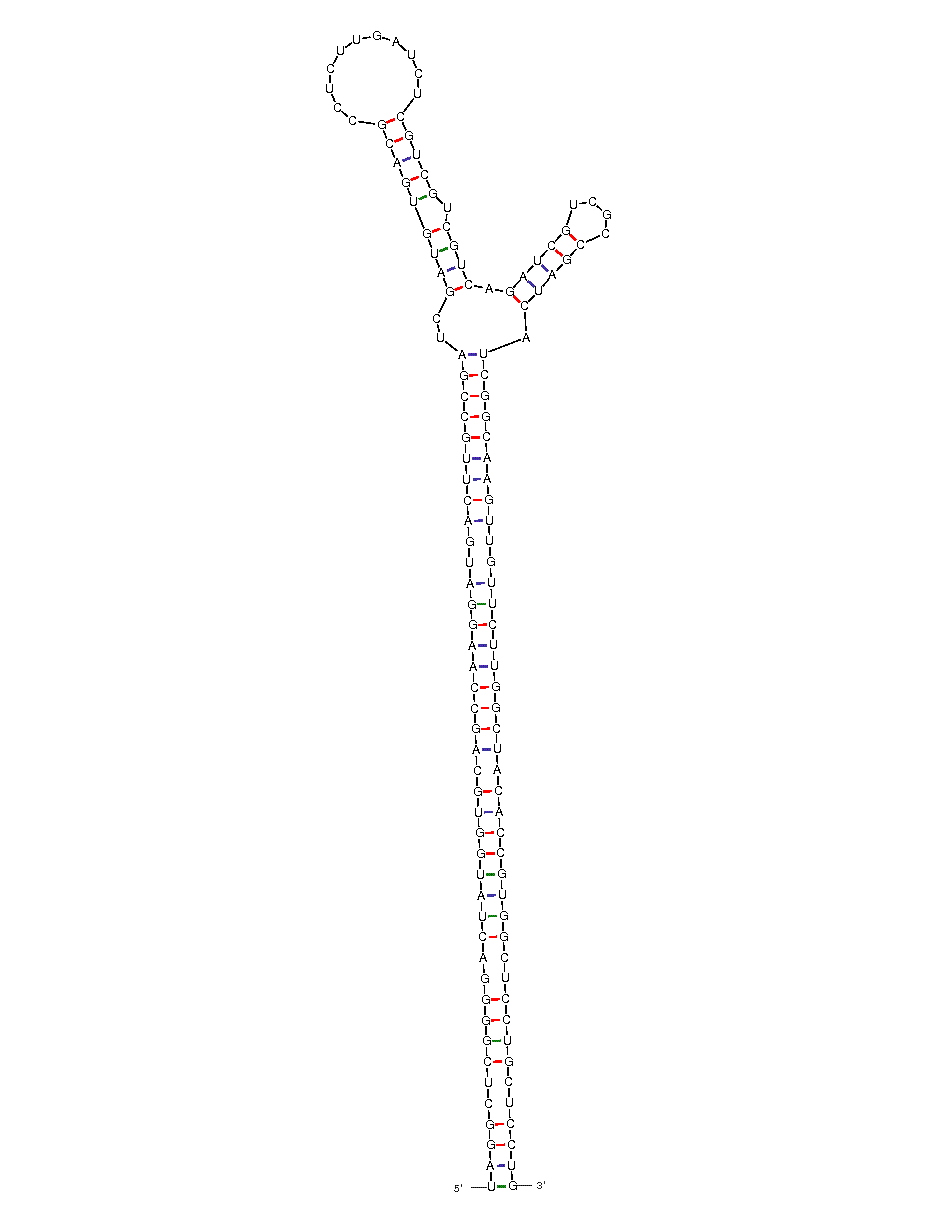


zma-miR393a


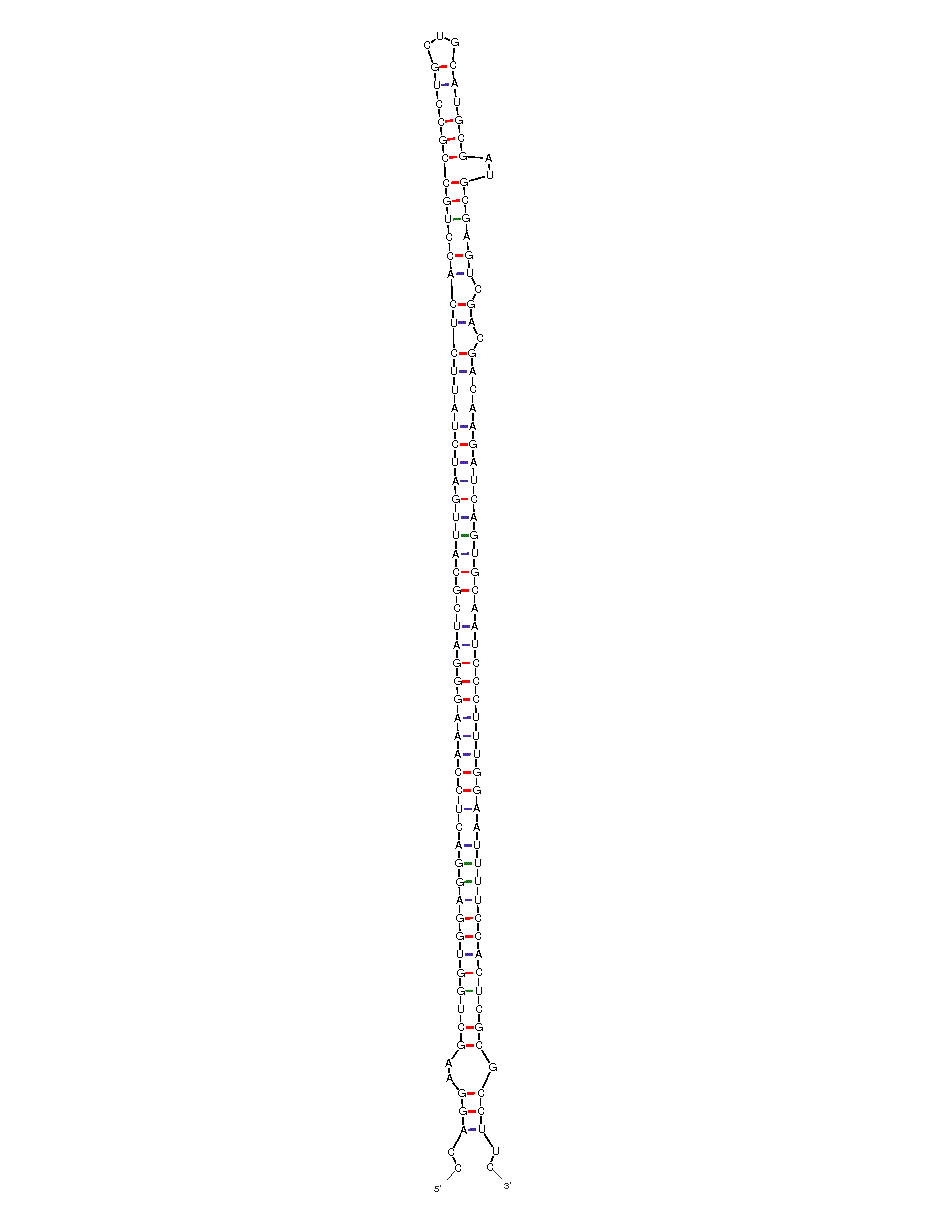


zma-miR396a


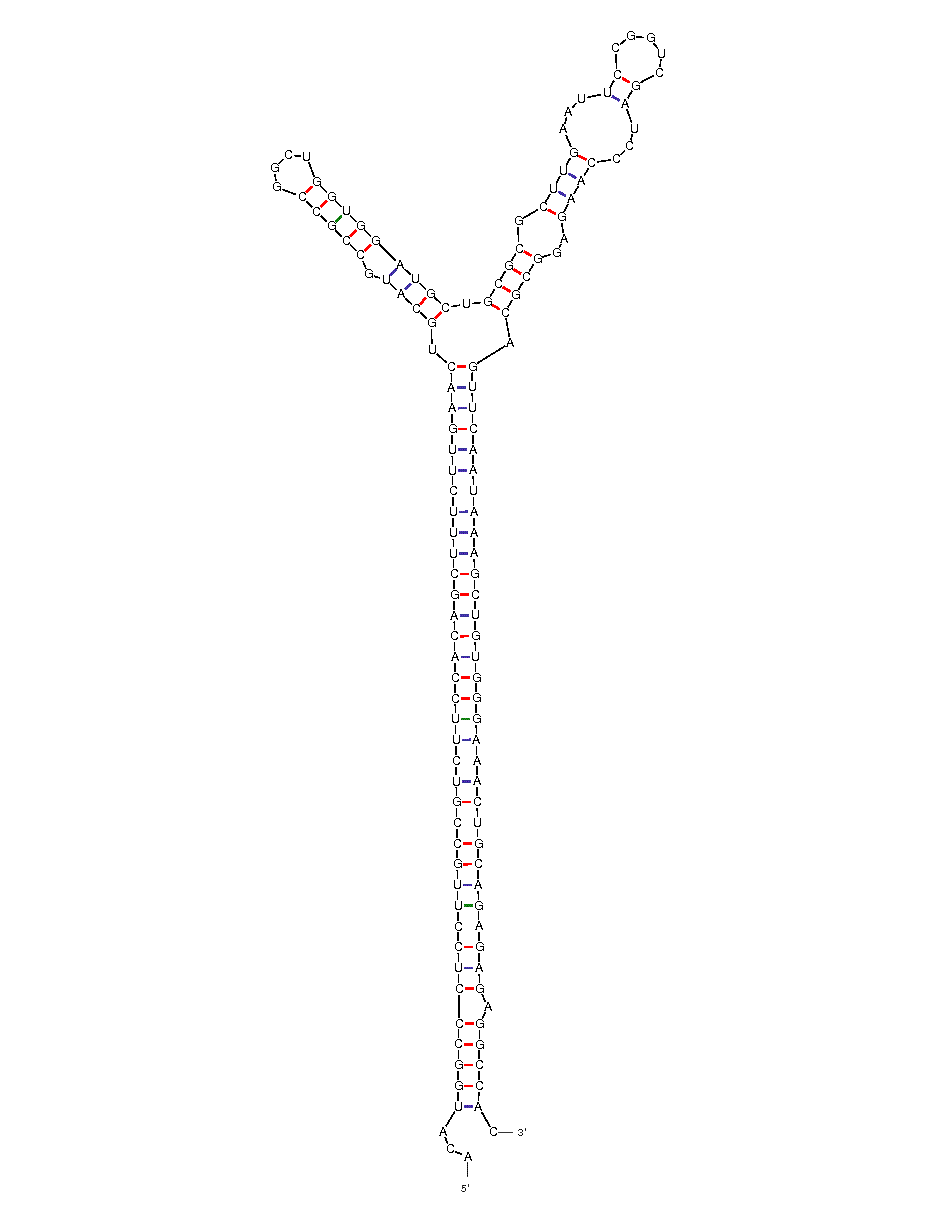


zma-miR397a


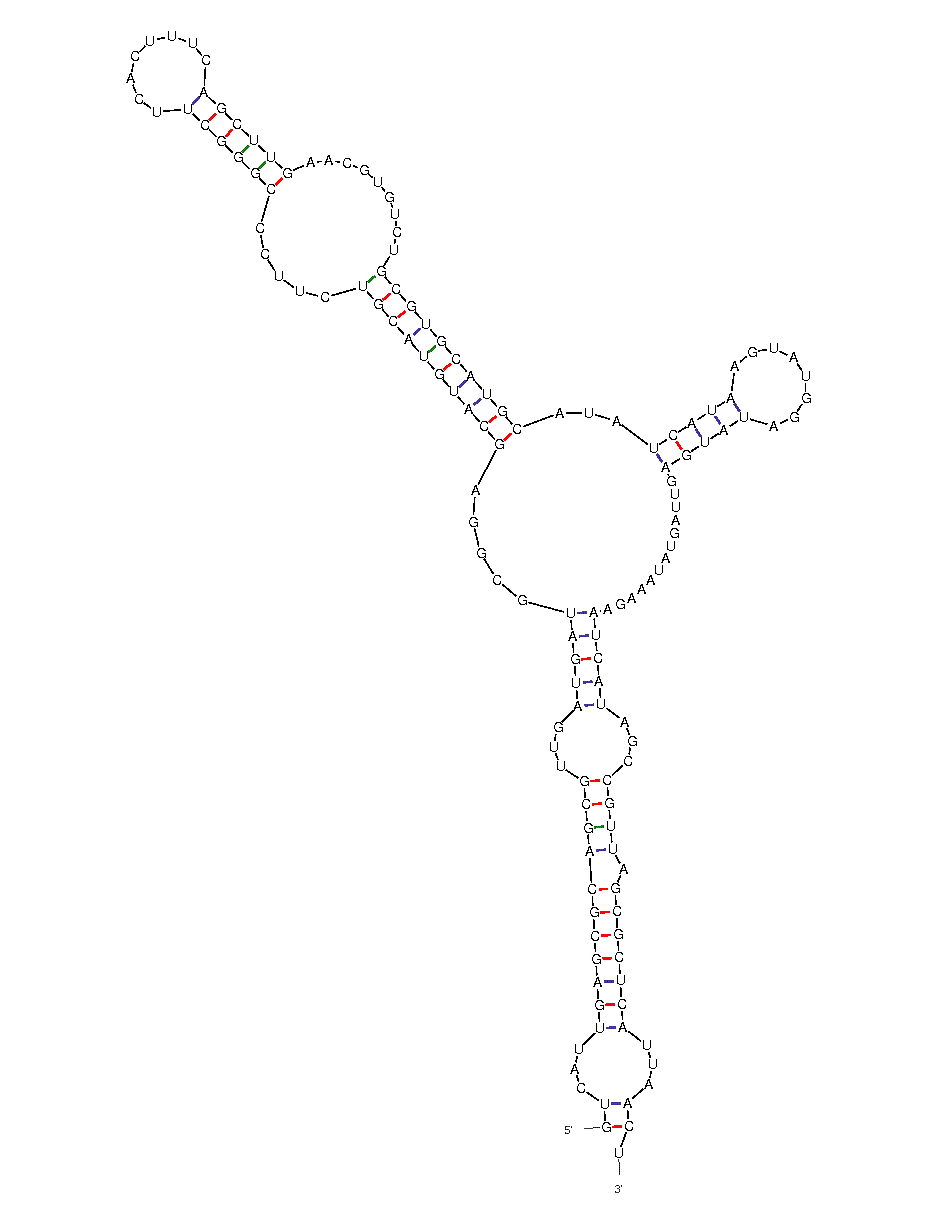

Supplement: S1 Fig — (DOCX) [file pone.0125800.s001.docx]
